# Opsonins and Dysopsonins of Nanoparticles: Facts, Concepts, and Methodological Guidelines

**DOI:** 10.3389/fimmu.2020.567365

**Published:** 2020-10-12

**Authors:** Emanuele Papini, Regina Tavano, Fabrizio Mancin

**Affiliations:** ^1^Department of Biomedical Sciences, University of Padua, Padua, Italy; ^2^Centre for Innovative Biotechnological Research, University of Padua, Padua, Italy; ^3^Department of Chemical Sciences, University of Padua, Padua, Italy

**Keywords:** nanoparticle corona, nanoparticle proteome, nanoparticle phagocytosis, opsonin, dysopsonin, nanoparticle stealthing polymers, innate pattern recognition molecules, complement cascade

## Abstract

Understanding the effects mediated by a set of nanoparticle (NP)-bound host biomolecules, often indicated with the *umbrella* term of *NP corona*, is essential in nanomedicine, nanopharmacology, and nanotoxicology. Among the NP-adsorbed *proteome*, some factors mediate cell binding, endocytosis, and clearing by macrophages and other phagocytes (opsonins), while some others display few affinities for the cell surface (dysopsonins). The functional mapping of opsonins and dysopsonins is instrumental to design long-circulating and nanotoxicologically safe next-generation nanotheranostics. In this review, we critically analyze functional data identifying specific proteins with opsonin or dysopsonin properties. Special attention is dedicated to the following: (1) the simplicity or complexity of the NP proteome and its modulation, (2) the role of specific host proteins in mediating the stealth properties of uncoated or polymer-coated NPs, and (3) the ability of the innate immune system, and, in particular, of the complement proteins, to mediate NP clearance by phagocytes. Emerging species-specific peculiarities, differentiating humans from preclinical animal models (the murine especially), are highlighted throughout this overview. The operative definition of opsonin and dysopsonin and the measurement schemes to assess their *in vitro* efficacy is critically re-examined. This provides a shared and unbiased approach useful for NP opsonin and dysopsonin systematic identification.

## Introduction

In this review, we summarize the present-day knowledge concerning host proteins able to up- or downmodulate the capture of nanoparticles (NPs) by phagocytes and other cells. In doing this, we also aim at challenging too easy, simplified, straightforward, yet quite widespread, conceptions of the interactions of NPs with host molecules, mostly based on mass spectrometry (MS)/omics shot-gun analyses. Eventually, we propose methodological and conceptual guidelines for a more effective research in this field.

In the section “Introduction,” we outline the historical emergence of the NP opsonin and dysopsonin concepts (section “The Discovery of Opsonin and Dysopsonin Activities Against Nanoparticles in Serum”), critically review the popular paradigm of the “NP corona,” and discuss a more comprehensive and balanced view of the interactions between host molecules and NPs (section “The “Nanoparticle Corona” Paradigm and Its Limit”).

In section “Re-examination of the Complexity of the NP Proteome Composition,” we address major methodological and conceptual issues relevant to assess the composition of the whole set of proteins binding to NPs. In particular, we highlight the pitfalls which may distort our view of the complexity of such phenomenon (section “Factors Overestimating the NP Proteome Complexity”). A milestone example is provided by the thoroughly discussed case of the NP proteome formed in bronchoalveolar lavage fluids (section “The Case of Bronchoalveolar Lavage Fluid-Derived NP Proteome”). Eventually, we show how the complexity of the set of NP-bound proteins can be strongly influenced by the nanosurface availability, in a given protein solution (section “The NP Proteome as a Function of Nanosurface Availability”).

In section “Influence on NP–Cell Interactions of specific NP-Bound Proteins,” we review all the studies where defined host proteins have been proposed to display opsonic or dysopsonic activities and critically analyze the supporting evidence. This somehow provides a first overview, although still partial, on the possible major actors involved in the opsonin/dysopsonin equilibrium on NPs. Lipoproteins and apolipoproteins are discussed in section “Lipoproteins, Apo B100, Apo E, Apo A4, Apo C3, and Apo H,” major proposed dysopsonins are treated in section “HRG, clusterin and HSA”. Immune agonists are reviewed in section “Complement C3-derived Opsonins, C1q, MBL, Properdin, IgG, SP-A, and SP-D”.

In section “Methodolgical Approaches to Identify NP Opsonins and Dysopsonins,” we list and critically evaluate the different methodologies applied in the present research to identify NP opsonins and dysopsonins, including cutting edge *in silico* approaches (section “Experimental Criteria”) and propose a conceptual frame useful to measure, without bias, opsonin and dysopsonin activities (section “Minimizing Ambiguities in Attributing Opsonin or Dysopsonin Properties to NP-Bound Proteins”).

In section “Opsonin-Dysopsonin Balance on Nanoparticles and Its Tilting by Complement,” we eventually present our view on the dynamic interplay between the different host-derived proteins interacting at the bio-nano interface. This model distinguishes two separate aspects: the passive interaction of some host proteins with NPs, largely governed by thermodynamic parameters, and the active, catalytically driven, recruitment of the major complement opsonins.

### The Discovery of Opsonin and Dysopsonin Activities Against Nanoparticles in Serum

The basic notion that host proteins can adsorb on nanoparticles (NPs) influencing their bioactivity has been present in literature for at least 30 years. Liposomes with different compositions and surface physicochemical features, which may be considered the prototypes of NPs, were first shown to selectively bind serum proteins able to influence their capture by phagocytes (1, 2). These studies and other related literature of the time clearly indicated that binding of plasma proteins to liposomal and polymeric NPs could be specific, depending on their surface chemical–physical properties. Recruited proteins were shown to dictate the biological fate of NPs, first of all endocytosis by phagocytes, and to represent a major aspect of NP host interaction, pharmacokinetics, and tissue targeting. In particular, Scieszka and Cho (4) early demonstrated that human serum busted the capture of nude liposomes by major blood professional phagocytes, the polymorphonuclear granulocytes (PMNGs), compared with the no-protein media. Notably, this effect was heat sensitive, being reduced at 56°C, this property is diagnostic of complement-mediated NP opsonization (3). A general overview of the physiological importance and of the molecular mechanisms of NP internalization by phagocytes and non-phagocytes is given in the Box 1.

**Box 1.**

The different ways phagocytes and non-phagocytes deal with NPs and the implications for the pharmacological outcomes of NPs. The distinction between phagocytes (or professional phagocytes) and non-phagocytes is based on the presence in the first cell category of an internalization mechanism termed *phagocytosis*, absent in other cells. Phagocytosis relays on large sub-membrane cytoskeletal rearrangement, allowing the engulfment of particles normally in the micrometer range (microparticles), like bacteria. However, also nanometer-size viruses (nanoparticles) can be cleared by phagocytosis, possibly in aggregated state (e.g. immune complexes with specific antibodies; or due to surface clustering after binding to membrane receptors). Phagocytosis is typically performed by blood-circulating myeloid leukocytes, as polymorphonuclear granulocytes and monocytes, able to migrate in inflamed tissues. In addition, a set of tissue resident macrophages, overall forming the so-called RES or Reticular Endothelial System, and in particular the liver Kupfer cells and the splenic macrophages, can capture blood micro or nano particulates via phagocytosis. Liver and spleen phagocytes, and in some animals also lung macrophages, are mostly involved in the blood clearance of micro-nanoparticles with pharmacological medical function. Phagocytes associated to mucosal epithelia, such as bronchial-alveolar macrophages are especially relevant for clearance of inhaled NPs. Macrophages in liver are mostly responsible for the short blood half-life of these carriers, unless they are effectively modified to be “stealth”, or *not intercepted by phagocytes*. The systemic activation of phagocytosis in blood is, on the contrary, excepted to determine adverse proinflammatory or pro-coagulant reactions and may contribute to HyperSensitivity Reactions upon NP administration.

Phagocytosis and clearance of NPs is functionally linked to the action of set of receptors, selectively expressed on phagocytes, which, once occupied by their ligands, indeed activate the phagocytosis mechanisms. FcRs and C3 receptors are the major responsible for the phagocytosis of particulates or immune-complexes decorated by Ig and complement derived opsonins C3d/C3bi. Several other membrane proteins expressed on phagocytes can bind to innate collectins (like MBLs, ficolins, C1q) or to molecular patterns present on microbes, altered cellular and proteins (e.g. scavenger receptors). Phagocytes are hence part of innate immune recognition and can also mediate elimination of particulates targeted by adaptive immunity (e.g. antibodies). Phagocytosis is a way to intracellularly confine potentially dangerous materials and microbes and, possibly, degrade and/or kill them.

Non-phagocytic cells are normally unable to activate the endocytosis of large microparticles or nanoparticles aggregates, since they only display what is called pinocytosis (cellular *drinking*), as opposed to phagocytosis (cellular *eating*). In pinocytosis, the invaginated membrane-bound vescicles have defined and small diameters which determine the cut-off or the dimension of the object to be internalized. For example, clatrin-mediated endocytosis has a cut off around 100-120 nm, while caveolin-dependent endocytosis has a cut off of 40-90 nm. Several other pinocytic mechanisms are differently expressed in cells but rarely they can support the same ability, displayed by professional phagocytes, to engulf large particulates. A good example of the potential importance of nanoparticles capture by non-phagocytic cells is the endotheliocyte: here the internalization via clatrin mediated or caveolin mediated pathways has limited impact on NP blood half-life, due to the their much-reduced efficacy compared to macrophages capture rate and capacity (eg. Liver Kupfer cells). However, endothelial cell endocytosis may be critical for the extravasation (transcytosis) and the reaching by the NPs of their final target. This is particularly true for endotheliocytes of the Blood-Brian Barrier (BBB).

In conclusion, binding and internalization by phagocytes antagonize the NP action, mostly affecting their pharmacokinetics. This may also give rise to adverse effects. In this context, the binding of an opsonin is to be considered a critical event affecting long-circulation and biocompatibility of the nanoformulations. On the contrary, for non-phagocytic cells, and especially for endotheliocytes, the binding to a NP of ligands which can be internalized by receptor mediated endocytosis (e.g. HDL or transferrin), not necessarily is a negative pharmacological event and maybe, in some cases, even desirable.

To prevent these adverse effects, pharmacologists soon developed specific liposome compositions and coatings, first of all conjugation with polyethylene glycol (PEG) or block copolymers like poloxamine-908, interfering with host protein adsorption on NPs (4, 5). The passivation of polystyrene (PS) and gold particles with these polymers was shown to decrease the binding of those serum opsonins favoring liver clearance by macrophage Kupffer cells and allowed the adsorption and action of specific, although not precisely identified, serum proteins with dysopsonic activities (6, 7). Among these, a protein with a Mw of >100 kDa was shown to be the major serum dysopsonin for PS NPs coated with poloxamine-908. In seminal studies dated back to the middle end of the 1990s of the past century, the first proteomic analysis was performed on liposomes, poly(D,L-lactic acid) (PLA), and poly(lactic-co-glycolic acid) (PLGA) NPs using, at the time, advanced techniques like the 2D gels and mass spectrometry (MS). The goal of these investigations was establishing a functional correlation between specific proteins bound to nude or polymer-coated NP and their phagocyte clearance efficacy (8–10). Leroux et al. (8) found that plasma proteins are responsible for an increased uptake of nude PLA NPs by human monocytes, while for a decreased uptake by non-phagocytic lymphocytes, *in vitro*. Moreover, PEGylation of these particles inhibited their uptake by all cells in the presence of plasma proteins, and such *stealth* effect was tentatively ascribed to the decreased absorption of apolipoprotein opsonins compared with their nude versions. Alleman et al. (11) observed a Ca^2+^-dependent enrichment of complement protein C3 on nude PLA NPs after incubation in serum and proposed a possible activation of complement mediated by immunoglobulin G (IgG; classical pathway), also abundantly observed on the NPs, so pointing the attention on complement factor 3 (C3)-derived opsonins as major players. Gref et al. (9) correlated the uptake of PEG-coated PLA, PLGA, and poly(varepsilon-caprolactone) (PCL) NPs by PMNGs in human plasma *in vitro* with PEGylation degree and protein adsorption. The total amount of plasma proteins absorbed on these NPs inversely correlated with PEG density grafting. Apolipoproteins and immunoglobulins were identified as possible major actors in regulating NP phagocytosis. The binding of both Apo C3 and Apo J, or clusterin, to PLGA NPs was shown to be drastically reduced by PEGylation (10).

Later on, it became clear that immune recognition systems may be critically involved in the binding of active triggers to PEG and other coats: for example, pre-existing anti-PEG antibodies [see, among others, (12)]. Also, lectins and other innate immune pattern recognition molecules (PRMs) were shown to bind to NPs and also to polymeric coats (13). Complement activation by the classical and the lectin pathways on the NP surface is often the result, with inflammation and phagocytosis. So, while the non-specific interaction and adsorption of proteins to NPs is governed by the laws of thermodynamic and electrostatics, it became evident that also the binding *via* specific binding sites, evolved in biological beings to monitor non-self or abnormal surfaces typical of microbes, or pathogen-associated molecular patterns (PAMPs), and of damage-associated molecular patterns (DAMPs) is critically involved in major biological effects of NPs (14–16).

Hence, the picture emerging at the beginning of the century, was that several serum proteins, could bind to NPs affecting their biological fate, favoring or reducing their capture by cells of the immune systems, or influencing their internalization in the target tissues.

### The “Nanoparticle Corona” Paradigm and Its Limit

**FIGURE 1:**
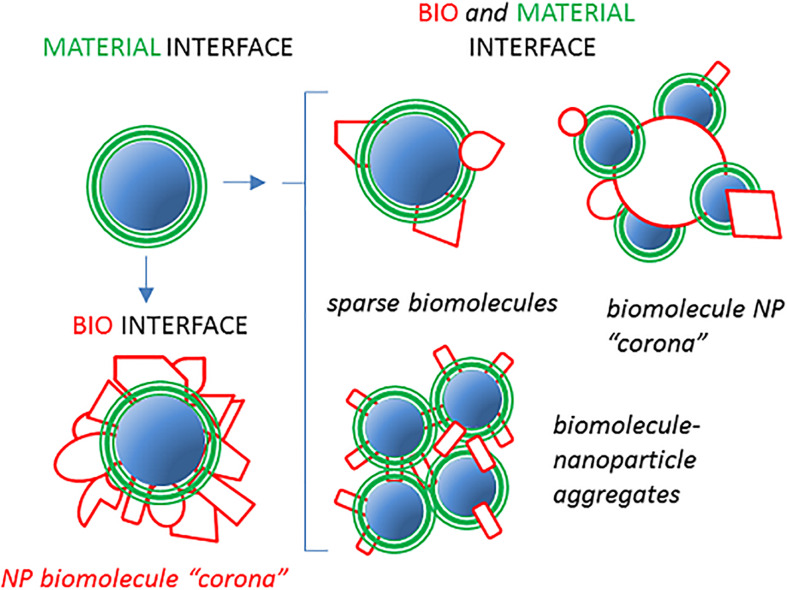
Nanoparticle (NP)–host serum protein complexes may assume different configurations. A pristine material displaying a defined interface with its environment (green) can be assumed to be coated with an almost continuous layer of host proteins (red) when introduced in serum/plasma or BALF. This phenomenon corresponds to the original view of the so-called NP “hard” corona creating a new biointerface, “seen” by cells (23). Alternative configurations, represented on the left, may result in partial coverage of the NP material by fewer proteins with new biological properties mixed with the original material coat exposure. In addition, NP aggregates or inverted situations in which a large biomacromolecule is surrounded by smaller NPs could generate situations in which the concept of corona may be misleading or inappropriate.

A generalized approach to characterize the composition and the function of NP-bound host proteins (and other biomolecules) emerged in the last decade thanks to comprehensive omics methodologies, such as the *shot-gun*. These studies prompted the formulation of the “NP protein (or biomolecule) *corona*” paradigm (17–22). Hundreds of polypeptide types have been reported to bind in different proportions to nanomaterials in host fluids and suggested to change the major physical–chemical, pharmacological, and biocompatibility features of NPs. The term *corona* originally evoked an almost continuous biomolecular interface between NPs and the host milieu, shielding the pristine nanomaterial surface and therefore creating new biological properties (23). However, subsequent research made the *corona* notion unfit to represent the whole range of possible NP–host molecule interactions. Indeed, a more heterogeneous spectrum of molecular assemblies/stoichiometry and architectures of the NP–protein complexes should be considered (Figure 1). For instance, it may be that few, but functionally effective, proteins are interspersed among or even buried below a predominant polymeric NP coat (24). It could also be that the reciprocal topology of NPs and proteins may be difficult to define (e.g., large host proteins, such as very low-density lipoprotein (VLDL) (40 nm), oligomeric surfactant protein-D (SP-D; 100 nm), or DMBT1^*gp*340^ (>200 nm) interacting with or even surrounded by a smaller “NP corona”) or that unstructured protein–NP aggregates are formed. To avoid bias and include any possibilities, instead of using the term *NP corona*, we here prefer the neutral expression of *NP proteome* to indicate the whole set of NP-associated proteins. We will use preferentially the term NP proteome in the rest of this review. Yet, also, the largely used term NP corona, in its modern conception of a complex biomolecular structure formed upon the exposition of a non-natural surface to biological fluids, maintains its validity.

Evidence support that the composition of the NP proteome, considered as a whole, is important to determine the biological action of NPs [see, for example, (25), and references quoted therein]. However, it would be useful and mostly desirable to understand whether and which defined proteins, assembled with NPs, exert a specific and dominant modulatory action on NP phagocyte clearance and cell targeting. Such molecular identification is an obliged step for a focused screening of new NP surfaces allowing the best design of stealth and biosafe coatings.

Despite extensive research, the clear-cut mechanistic demonstration of the effect of well-defined components on cell–NP interaction is limited. To assess this issue, we will here focus on recent literature in which the hypothetical role of specific NP-bound host proteins (serum/plasma and bronchial alveolar fluid) in cell association was functionally tested. In this analysis, we will also focus on the impact which may be derived from variations of the NP surface/protein concentration ratio on the composition and complexity of the NP proteome. We will pay special attention to data supporting the role of single proteins in decreasing (dysopsonin) or favoring (opsonin) the capture of NPs by macrophages and other phagocytes.

## Re-Examination of the Complexity of the NP Proteome Composition

### Factors Overestimating the NP Proteome Complexity

Understanding the role played by each single component among the NP proteome in cell interactions, and, in particular, with immune cells, may seem a very difficult task due to the high number (hundreds) of different polypeptide types which have been found to be associated with different NPs in variable proportions (19). A predominant popular view maintains indeed that the NP-associated protein ensemble is very complex in terms of chemical composition and, hence, displays very complex biological effects.

However, based on simple geometric considerations, it seems that the estimated number of total polypeptide molecules per NP measured by shot-gun proteomics is, in many cases, much larger than the amount that could be accommodated on its surface (26). For example, the surface of a 20-nm diameter NP can host no more than 60 (and likely less) globular proteins with 5 nm diameter. Accordingly, it was experimentally found that one single 26-nm-diameter SiO_2_-NP maximally binds only about 27 fibrinogen (MW 340 kDa) or 30 HRG (histidine-rich glycoprotein; MW 70 kDa) or 47 kininogen 1 (Kin-1) molecules (MW 110 kDa) (27). Consistently, several, if not the majority, of the identified polypeptides in various NP-proteomes should be and are indeed found to be moderately or strongly sub-stoichiometric (19, 28, 29).

The presence of sub-stoichiometric proteins in the NP-proteome is somehow surprising but can find different justifications. A first possible reason is the presence of NP subgroups within the same produced population with differentiated protein compositions due to variations of NP physicochemical features around the mean value (size, zeta potential). The perception of a strong heterogeneous composition of the NP proteome may also be derived from the improper equivalence between the terms *polypeptide* and *protein* made in data representations, which overlooks the quaternary structure of proteins and specific protein–protein interactions. For example, one C1 molecule, the first complement factor (C1q) of the classical cascade, is made by six C1qA, six C1qB, six C1qC, two Cr, and two Cs polypeptides. Similarly, one IgM molecule, able to bind C1, comprises 10 heavy chains, 10 light chains—lambda or k isoforms—and one J chain. Hence, if we imagine a theoretical situation in which one single IgM/C1 complex is present on one NP, this will correspond to 10 different polypeptide types for a total of about 43 polypeptide molecules per NP. Informatics analysis restitutions also over-represents antibody complexity since all the different variables, D and J, and constant segments contributing to the creation of different heavy and light chains of one specific immunoglobulin molecule, due to somatic recombination, are encoded by 148–171 genes in the human genome but are annotated as distinct proteins in data base (e.g., see Uniprot data base https://www.uniprot.org/). Even more importantly, the apoprotein part of lipoproteins, a major heterogeneous component associated with NPs (30, 31), comprises tens of different polypeptide types, often in sub-stoichiometric ratios (32). For instance, recent global proteomics analysis documented up to 90–100 polypeptides differently distributed in various high-density lipoprotein (HDL) size populations (33). Therefore, the NP proteome apparent complexity in serum/plasma might in part mirror the intrinsic proteome heterogeneity of NP-bound lipoproteins.

Eventually, the high number of components found may also be due to residual contaminants after washing procedures with no functional meaning (34), possibly favored by NP aggregations.

What emerges from this analysis is that the NP proteome is not necessarily a complex and elusive ensemble of hundreds of proteins. We will see in the following paragraphs that, depending on NP nature, size, and on the experimental conditions, relatively simple NP proteomes may form, allowing in principle a relatively straightforward prediction of their properties.

### The Case of Bronchoalveolar Lavage Fluid-Derived NP Proteome

A special, but physiologically central, case of NP–host protein interaction occurs when inhaled NPs enter the surfactant film covering the respiratory mucosae. Here, a dominant role in NP coating is also believed to be uniquely played by the lipid component of the surfactant film, with configurations which may strongly depend on the hydrophilicity or the hydrophobicity and lipid solubility of NP formulations [see (35) and other references therein quoted]. The first comprehensive analysis, based on shot-gun proteomics in porcine bronchoalveolar lavage fluid (BALF) from slaughtered animal lungs suggested a complex composition (about 400 different polypeptides) of the proteome associated to PLGA-, PEG-, and lipid-coated NPs (36). However, in this study, the first 25 most abundant polypeptide types bound to any of the used NP types, roughly representing the most part of the stoichiometric components (of total 376 polypeptides), accounted for ∼65% the “NP proteome” mass. Even more significantly, within this pool, cytoskeletal or cytosolic proteins, likely contaminants released by damaged cells, accounted for ∼48% of the total proteome mass, while the collectin surfactant proteins-A (SP-A), the major protein component of the pulmonary surfactant, represented about 2–4% of the total proteome mass.

This odd prevalence of intracellular proteins raises some concern on the real physiological composition of the BALF-derived NP proteome as here characterized. Indeed, subsequent investigations, using human BALF from patients affected by pulmonary alveolar proteinosis (PAP) as a model, supported a different scenario (37). These authors pointed out that the collectin SP-A and other few major surfactant proteins accounted for a large fraction of total bound proteins and where consistently, stably, and abundantly associated with NPs, determining a relatively simple composition of the NP proteome formed in this host fluid.

It could be that part of discrepancies in these two studies derives from the different species and BALF isolation procedures (lung washes from slaughtered pigs versus therapeutic lung washes from live humans). Still, these studies highlighted the major role of innate PRMs in the interaction with inhaled NPs: the two collectin SP-A and SP-D and the product of deleted in malignant brain tumor 1 (DMBT1) gene. These are also major constituents of the NP proteome in bronchoalveolar fluid. The DMBT1 gene, thanks to alternative splicing mechanism, encodes for a family of glycoproteins involved in innate immunity and tissue repair at the mucosal level (38). The largest of the produced polypeptides (340 kDa), called salivary agglutinin/glycoprotein-340/DMBT1, secreted into bronchoalveolar surface lining fluid or in the saliva, contains 14 scavenger receptor cysteine-rich (SRCR) domains. It can agglutinate several Gram-negative and Gram-positive bacteria, interact with SP-A and SP-D and other immune agonists like C1q, sIgA, and lactoferrin and activate the complement cascade *via* the lectin pathway.

Also, in the case of BALF, NP proteome compositions might be relatively simple, with a small number of predominant host proteins dictating the NP fate.

### The NP Proteome as a Function of Nanosurface Availability

A quite obvious but often neglected consideration is that the ratio between the NP surface available and the concentration of potential NP binders influences the composition and the degree of complexity of the NP proteome. Indeed, it is likely that if the total surface is limited, because of low NP concentration, host-biomolecules having optimal affinity/concentration characteristics will compete more effectively for NP binding. This fact can potentially allow the detection of functional effects mediated by specific proteins at low NP concentrations, which did not reveal at high NP concentrations (22, 27, 29).

Fedeli et al. (27) actually showed that the composition of the proteins bound to SiO_2_-NPs strongly depends on the NP concentration, and hence on the NP surface/serum protein ratio. In contrast with the main common view maintained in the field that hundreds of polypeptide types are found associated to a typical NP, below a certain NP concentration (∼40 μg/ml) the NP proteome of 26 nm diameter amorphous SiO_2_-NPs in human plasma was largely formed by HRG (30 molecules/NPs), binding to silica with high affinity (*K*_d_ = 2.4 nM), and to a minor extent by the homologous protein high molecular weight kininogen (HMWK) (6 molecules/NP; *K*_d_ = 4 nM). On the contrary, above ∼40 μg/ml, a switch was observed in the NP proteome composition and other proteins were recruited forming a more complex proteome where fibrinogen, HDL and low-density lipoproteins (LDL), and IgG were major components. This switch can be explained assuming that human serum HRG is preferentially adsorbed at low NP doses; due to its high affinity, the silica surface, and that, after exhaustion of HRG consequent to NP concentration increase, space is left on the NP surface for the recruitment of other proteins with reduced affinity for silica. Accordingly, Francia et al. (29) showed that at low (12 mg/ml) serum content (i.e., high surface/serum ratio), HDL (Apo AI) was the major component associated to SiO_2_-NPs (300 μg/ml) followed by HGR (17.9 and 5.4%, respectively) while at high (62 mg/ml) serum content (i.e., low surface/serum ratios) HRG became the most abundant NP bound protein followed by Apo AI (9.5 and 7%, respectively). The HRG prevalence in conditions of nanosurface limitation allowed to evidence its dysopsonic activity (27). In fact, SiO_2_-NP macrophage capture is substantially inhibited below 40 μg/ml SiO_2_-NP concentration. Purified HRG used at doses resembling the ones present in human serum inhibits macrophages capture as well, while HRG-depleted serum resulted in more effective NPs uptake by macrophages, which was abolished by restoration with purified HRG. It is also intriguing to observe that HRG depletion leads to the major recruitment of another protein, HMWK, which is homologous to HRG but devoid of intrinsic dysopsonic effects.

**FIGURE 2:**
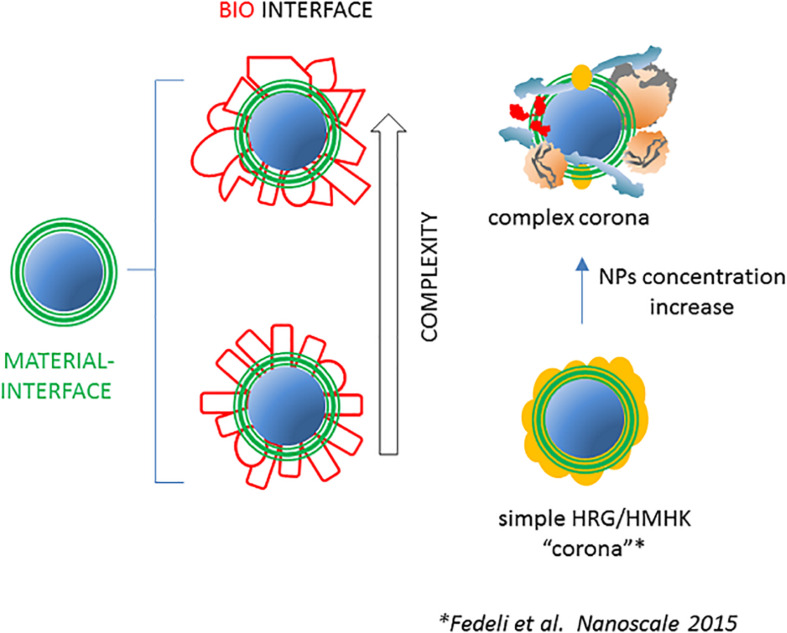
The NP-associated proteome may have various degrees of complexity. As represented on the left, the complexity of the set of NP-bound proteins after incubation with complex host fluids is assumed to be high in terms of numbers of different polypeptides and of total bound molecules. Evidence suggests that in some conditions, the NP proteome may be simpler, *i.e.*, formed by few molecular species in a more limited number. An example is represented on the right where the NP concentration decrease in human plasma, due to stronger reciprocal competition for the diminishing available surface, results in the conversion of a relatively complex and heterogeneous NP proteome into a simple and more homogeneous one, characterized by the prevalence of HRG and HMWK (27).

The above evidence proves that, at least in special NP/protein ratio conditions, or in special fluids (the BALF) and considering polypeptide structural assembly in proteins, the NP proteome may be indeed relatively simple and mostly represented by few stoichiometric protein types (Figure 2). Moreover, these data point the attention on the importance of NP dose and suggest that adsorption of proteins in conditions of high NP surface availability is less selective, since competition and reciprocal interference between serum proteins is less relevant. As a general trend, the composition of the NP proteome is predicted to be more heterogeneous at high (roughly mg/ml) NP concentrations (i.e., increased surface/serum concentration ratio) and tends to be less specific compared with serum composition, especially for highly absorbing nanomaterials like silica.

The above example is also interesting because it highlights a major difference between the human and the mouse serum. In fact, the transition from a simple HRG-enriched proteome to a complex fibrinogen/HDL-enriched one, observed at NP doses of >40 μg/ml SiO_2_-NPs in human serum, is not observed up to 400 μg/ml SiO_2_-NPs in mouse serum. This observation predicts that the amount of HRG in mice is much higher. This represents a case in which the NP-concentration dependence of the NP proteome is species specific due to the peculiar characteristic of the serum of this mammal compared with the human being. It is not impossible that other qualitative and quantitative differences in the serum composition differently modulate its biological effects (including phagocytes capture) in humans and major preclinical models. In line with this concept, the proteome associated to bare or PEGylated SiO_2_-NPs was found to be significantly different in human and mouse serum (27, 39).

## Influence on NP-Cell Interactions of Specific NP-Bound Proteins

**FIGURE 3:**
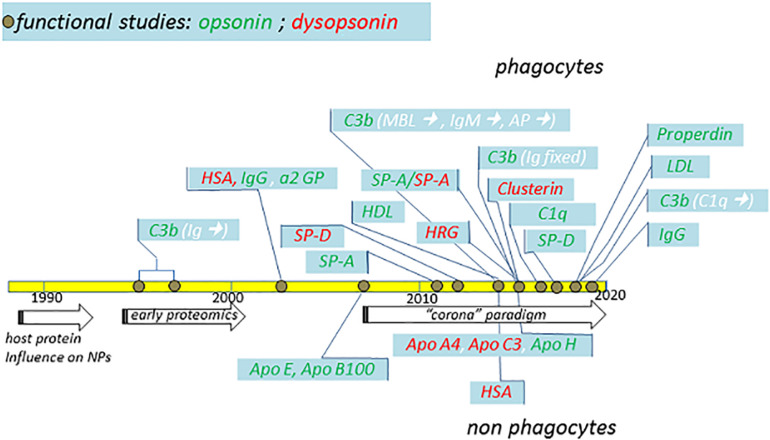
Schematic summary of major functional studies in the last 32 years suggesting the identification of specific NP opsonins and dysopsonins. The indicated studies (detailed in the text) and their publication years are compared with major general achievements (arrows) in the fields. Dysopsonins are in red, while opsonins are in green. In the case of C3b opsonin, the complement triggers are indicated in white within brackets. Studies performed using phagocytes are on top, while studies performed using epithelial or endothelial cells are on the bottom.

In parallel with the explosion of the *corona* idea, several studies identified protein agonists favoring or inhibiting the capture of NPs by phagocytes and non-phagocytes (Figure 3). However, it is important to note that the first evidence of a defined opsonin for phagocytes, complement C3b, was obtained in 1995–1997, immediately after the identification of the general phenomenon of host protein influence on NPs, and in concomitance with the early proteomic characterizations. Below, we review such evidences, also summarized in Tables 1, 2, and their strength, based on protein types or broad classes.

**TABLE 1:** Proteins with NP opsonin or dysopsonin properties in phagocytes.

Protein	NPs (∼ size)	Activity	Cellular model	Functional evidence	Relevant or critic notes	References
High Density Lipoproteins (HDL)	SiO_2_ (26 nM)	Opsonin	Macrophages differentiated from human blood monocytes	Effect of purified lipoprotein compared to human serum albumin (HSA)		Fedeli et al. (40)
Histidine Rich Glycoprotein (HRG)	SiO_2_ (26 nM)	Dysopsonin	Macrophages differentiated from human blood monocytes	Effect of purified protein compared to other purified plasma proteins/plasma depletion - reconstitution	Complement inhibited (citrated plasma)	Fedeli et al. (27)
Clusterin/Apo J	PEG or PEEP coated polystyrene NPs (100 nm)	Dysopsonin	Mouse RAW264.7 macrophagic cell line	Effect of the purified protein compared to no protein medium and purified HSA	Control HSA used at non-physiological dose; human protein versus murine cells	Schöttler et al. (45)
	Ag-NPs (10 nm)	Dysopsonin	Macrophages differentiated from the human monocytic cell line THP-1	Effect of the purified protein compared to no protein medium and purified HSA	Control HSA used at non- physiological dose	Aoyama et al. (47)
	SiO_2_-NPs (70 nm)					
Human serum albumin (HSA)	Differently charged polystyrene (1 μM)	Dysopsonin	Human dendritic cells	Effect of purified human serum albumin compared to purified IgG and α2 GP and to no protein	Microparticle	Thiele et al. (50)
CO3b/iC3b	ORMOSIL PMOXA NPs (100 nm)	Opsonin	M-CSF differentiated macrophages from human blood monocytes, human blood monocytes and PMNGs	Ca^2+^ dependence/complement factors depleted sera – reconstitution/complement neutralizing antibodies/statistical correlation	C1q directly bound to NPs triggers complement	Tavano et al. (28)
	Dextran-coated SPIO-NWs (110 nM); LipoDox (100 nm, PEGylated liposomes); Onivyde (120 nm, PEGylated liposomes);SPIO Feraheme	Opsonin	Human macrophages		C3b/iC3b fixed on anti NPs “natural” IgG	Vu et al. (69)
	Poly (D,L-lactic acid)	Opsonin	Human monocytes	Ca^2+^ dependence		Leroux et al. (56)
	Iron Oxide Nano Worms	Opsonin	Mouse peritoneal macrophages, neutrophils, monocytes and lymphocytes; human neutrophils, monocytes, lymphocytes and eosinophils	Ca^2+^dependence/complement factors depleted sera – /complement neutralizing antibodies/Lectin inhibitory sugar	MBL triggered (mouse); MBL and AP triggered in human, sporadically also natural IgM triggered	Banda et al. (62)
						Wang et al. (81)
						Inturi et al. (63)
C1q	CMC-MWNT, Ox-MWNT (10–20 nm × 5–20 μM)	Opsonin	U937 cells and human macrophages	Effect of purified protein compared to no-protein media	purified subunits of C1q separately induce the same effect of entire protein	Pondman et al. (66)
Properdin	Carboxyl-methyl cellulose coated CNT	Opsonin	THP-1 macrophagic human cell line	Effect of purified protein compared to no protein medium	independent on complement activation	Kouser et al. (68)
IgG	SiO_2_-NPs (100 nm)	Opsonin	HEK-293T	Fc receptor overexpression	non-phagocytic cells expressing FcR as phagocyte model	Lara et al. (41)
	PLGA based NPs	Opsonin	mouse RAW 264.7 macrophagic cell line and CHO-K1	FcR negative and positive cell lines	murine phagocytes as FcR + cells and hamster non-phagocytes as FcR – cells; human plasma	Rezaei et al. (70)
	Polystyrene (1 μM)	Opsonin	Human dendritic cells	Effect of purified human IgG compared to purified HSA		Thiele et al. (50)
SP-A	Amine-modified cationic PS (100–200 and 500 nm)	Opsonin	Mouse alveolar macrophages and RAW 264.7 macrophagic cell	Effect of purified proteins compared to no protein media		Mc Kenzie et al. (73)
	Anionic PS NPs (100–200 and 500 nm)	Dysopsonin				
	Variously coated magnetite NPs (110–180 nm)	Opsonin	Murine alveolar macrophages	Effect of purified protein compared to BSA		Ruge et al. (74)
	Mannosilated PEG on PLGA/PLA NPs (140 nm)	Opsonin	Murine alveolar macrophages; TPH-1 macrophagic human cell line	Effect of purified protein compared to no protein	alveolar macrophages capture *in vivo*	Ruge et al. (77)
SP-D	CMC-CNT (10–20 nm × 5–20 mm)	Opsonin	U937 and THP-1 human cell line	Effect of purified protein compared to no protein		Pondman et al. (79)
	OxCNT (10–20 nm × 5–20 mm)	Dysopsonin				
	CMC-CNT (10–20 nm × 5–20 mm)	Opsonin	Murine alveolar macrophages and lung dendritic cells	Comparison of SP-D containing BALF with SP-D lacking one	capture *in vivo* using control or SP-D Knock Out transgenic mice	Kendall et al. (80)

*Studies which proposed the opsonin or dysopsonin nature of well-defined proteins are shown. The cellular models, major evidence provided and critical aspects are in brief reported. Dendritic cells, due to their close relation to macrophages are here included as phagocytes. Epithelial cells, used as platform to express typical phagocyte opsonin receptor (e.g., FcR), we considered model “phagocytes” and data were therefore here classified. HDL, High Density Lipoproteins; HSA, human serum albumin; HRG, histidin rich glycoprotein; PEG, poly ethylene glycol; PEEP, poly(ethyl ethylene phosphate); Ag-NPs, silver nanoparticles; IgG, immunoglobulin G; α2 GP, alpha 2 glycoprotein; ORMOSIL PMOXA NPs, Organically modified silica polymethyloxazoline nanoparticles; M-CSF, macrophage colony stimulatory factor; PMNGs, polymorphonuclear granulocytes; SPIO-NWs, superparamagnetic iron oxide – nano worms; MBL, mannose binding lectin; AP, Alternative Pathway of complement activation; C1q, complement factor 1 q; C3b, complement factor 3 b; IgM, immunoglobulin M; CMC-MWNT; carboxymethyl cellulose coated-multi wall nanotubes; Ox-MWNT, oxidized multi wall nanotubes; CNT, carbon nano tubes; Fc, fragment c of immunoglobulins; PLGA, poly lactic glycolic acid; PLA, poly lactic acid; BSA, bovine serum albumin; FcR, Fragment c receptor; SP-A, surfactant protein A; SP-D, surfactant protein D;CMC-CNT, carboxymethyl cellulose coated carbon nanotubes; OxCNT, oxidized carbon nanotubes; BALF, bronchial alveolar lavage fluid.*

**TABLE 2:** Proteins with NP “opsonin” or “dysopsonin” properties in non-phagocytes.

Protein	NPs (∼ size)	Activity	Cellular model	Functional evidence	Relevant or critic notes	References
Low density lipoprotein (LDL) Apo B100	SiO_2_-NPs (100 nm)	Opsonin	Human A549 cells/HEK-293T cells	LDLR downregulation (siRNA)/expression		Lara et al. (41)
Apo B100 Apo E	poly(ethylene glycol) polyhexadecylcyanoacrylate (PEG-PHDCA) NPs (135-171 nm)	Opsonin	Primary rat brain Endothelial cells	Effect of the purified protein compared to NPs alone; block by anti-LDLR mAb	5% FBS present in all conditions; no lipid component	Kim et al. (43)
						Kim et al. (44)
Apo A4	COOH modified polystyrene NPs (100 nm)	Dysopsonin	Human cancer cell line (HeLa)[Frame1] and primary human mesenchymal stem cells (hMSCs)	Effect of the purified Apo proteins compared to no protein media	Recombinant apo proteins separate from the lipid components	Ritz et al. (42)
Apo C3		Dysopsonin				
Apo H		Opsonin				
HSA	Dihydrolipoic acid-coated –QDs (5 nm)	Dysopsonin	Human cancer cell line (HeLa)	Effect of the purified protein compared to no protein		Treul et al. (51)

*Studies where the opsonin or dysopsonic action of defined proteins were proposed, in non-phagocytic cells. The term opsonin/dysopsonin is here borrowed from the phagocyte context, and indicates the action of proteins on NP pinocytotic internalization by non-phagocytic cells. LDLR, low density lipoprotein receptor; FBS, Fetal Bovine Serum; QDs, quantum dots; HSA, human serum albumin.*

### Lipoproteins, Apo B100, Apo E, Apo A4, Apo C3, and Apo H

Blood lipoproteins are major components associated to several NP types (22). Fedeli et al. (40) showed that purified human HDLs increase the capture of SiO_2_-NPs by human macrophages but not by human monocytes. In this study the exhaustion of HDL from the surface of SiO_2_-NPs above a critical NP dose also induced necrotic effects in macrophages. However, HDL role in the whole complex serum mixture is still hypothetical since no evidence from HDL-depleted sera was provided. Based on indirect evidence, LDL Apo B 100 was proposed to be critical for the uptake of SiO_2_-NPs *via* the LDL receptor (41). Although purified human LDLs were found to mediate the capture of SiO_2_-NPs by human macrophages, while not by human monocytes and non-phagocytic lymphocytes (27), similar effects were also displayed by several other SiO_2_-NP-associated serum proteins [HDLs, VLDLs, Kin-1, fibrinogen, IgG, and human serum albumin (HSA)]. When all these proteins were mixed, the lack of none of them resulted in loss of macrophage capture. This suggests that all these proteins (included LDL), although endowed with intrinsic pro-phagocytic affects in macrophages, are interchangeable in their function, being hence sufficient but not necessary to the pro-opsonic effect in macrophages. Ritz et al. (42) proposed that Apo A4 and Apo C3 counteract NP mesenchymal and cancer cell targeting, based on their presence in the proteome of COOH-derivatized PS NPs in human serum and on the ability of these purified apolipoproteins to induce a strong decrease of cell capture (80% inhibition) compared with bare NPs. However, the functional effect of lipid-free apolipoproteins is questionable since, in physiological conditions, Apo A4 and Apo C3 are part of VLDL, L(a), and LDL whole proteolipid complexes. This caveat does not apply to the proposed opsonin Apo H (also called beta-2-glycoprotein 1) which, despite its name, only partially adsorbs to lipoproteins and is largely free in serum and which, as purified agonist, was shown to induce a twofold increased cellular uptake of NPs compared with no-protein media conditions (42). Purified Apo E and Apo B100 have also been shown to improve the endocytosis of poly(ethylene glycol) polyhexadecylcyanoacrylate (PEG-PHDCA) NPs in primary rat brain endothelial cells (RBEC), compared with NPs only, *via* specific binding to cellular LDL receptors (43, 44).

### HRG, Clusterin, and Albumin

As anticipated above, Fedeli et al. (27) showed that HRG has dysopsonic effects on SiO_2_-NPs, based on several evidences. First, when the HRG amount in the NP proteome drops, due to NP dose increase and HRG exhaustion in plasma, NP capture by macrophages improves in parallel. Additionally, the single purified protein used at physiological doses was shown to act as dysopsonin, while the whole set of other major NP proteome components (HMWK, HDL, LDL, VLDL, IgG, fibrinogen, and HSA) used all together or in several combinations had no dysopsonic action. HRG-depleted plasma lost the HS antiopsonic effect at low NP doses, which was regained upon purified HRG reintroduction in the system. Indeed, HRG effectively competed also with fetal calf serum (FCS) proteins impeding their association to NPs, blocking the macrophages capture of NPs observed in this medium. However, experiments were performed in citrated plasma, where Ca^2+^-dependent complement pathway is inhibited and eliminated from the scenario (see below).

Special attention must be dedicated to clusterin since this chaperonin (also called Apo J) has been proposed to play a fundamental role in conferring the so-called stealth feature, or ability to avoid NP-clearance by capturing macrophages (45). Selvestrel et al. (46) already showed that, while HRG was the major protein associated to inorganic SiO_2_-NPs as discussed above, clusterin was the major protein bound to organically modified silica (ORMOSIL) NPs after incubation in human serum. Using PEG or poly(ethyl ethylene phosphate)-coated PS NPs, Schöttler et al. (45) proposed that the ability of these polymers to confer NP stealth characteristics was not directly due to the polymer itself but, rather, to its ability to effectively bind and recruit clusterin on NPs. This would correspond to a paradigm shift since clusterin as a dysopsonin would have the final responsibility for the macrophage escape ability of polymer-coated NPs. The possibility of a specific affinity of clusterin for NP PEG coating is however not supported by other studies. Early studies already discussed reported that clusterin binding to PLGA NPs in human plasma was totally abrogated after PEGylation of these NPs (10). In several other cases, as the ORMOSIL NPs above cited, clusterin binds to NPs also in the absence of a PEG coating.

Aoyama et al. (47) found that clusterin is a major component also on uncoated silver and silica NPs. In both studies, purified human clusterin demonstrated some intrinsic stealthing power at physiological serum concentration (50 μg/ml) compatible with whole serum effect. However, control experiments with the major plasma protein albumin, to show clusterin specificity, were not homogenous since being performed at a dose (50 μg/ml) that is ∼1000 times reduced compared with its physiological serum concentration (∼60 mg/ml). Simon et al. (48) showed that clusterin binding to PEG-coated PS NPs was inhibited by pretreatment of human plasma and serum at 56°C and that this resulted in a higher binding of NPs to murine RAW264.7 macrophages, in line with the major antiopsonic effect of clusterin. However, although purified clusterin reintroduced in preheated serum was found to partially bind back to NPs, the restoration of stealth effect was not assessed. Interestingly, in the same study, the authors also showed that human serum/plasma increased the capture of hydroxyethyl starch (HES) nanocapsules by macrophages and that this paralleled the deposition of complement protein C3 on NPs. Thermal (56°C) serum treatment strongly reduced both complement protein NP association, in agreement with heat sensitivity of the complement cascade (49), and NP macrophage capture. Since serum heat pretreatment also strongly abolished the binding of clusterin to NPs, a phenomenon which should result in a stronger cell uptake, data suggest that the loss of NP opsonization by C3 was functionally predominant on the parallel disappearance from the NP surface of the supposedly stealthing/dysopsonin clusterin. At the end, clusterin was apparently neither necessary nor sufficient to mediate the stealth action of serum in this case, and its presence on NPs was not useful to predict phagocyte capture evasion. On the contrary, this study supported a much more solid correlation between murine macrophage capture and complement activation. Consistently, Tavano et al. (28) observed strong and similar clusterin binding to uncoated PEG, and polymethyloxazoline (PMOXA)-coated ORMOSIL NPs in human serum, but this medium improved macrophage capture of polymer-coated NPs and not of the bare ones. Moreover, this study showed that Ca^2+^ sequestration selectively and totally abrogated complement activation and phagocyte capture of coated NPs but did not grossly affect the abundance of clusterin in the NP proteome. Eventually, comparing the effect of different sera from different donors, macrophage capture efficacy positively statistically correlated with the extent of C activation and C3b NP opsonization but did not negatively correlate with clusterin presence on NPs.

The presence of serum dysopsonin antagonizing opsonins was suggested quite early (6). Being albumin the most abundant serum protein, studies focused on this protein. Thiele et al. (50) showed that purified HSA strongly decreases the phagocytosis of PS microparticles with various surface charges by dendritic cells and also antagonized the opsonic action of IgG and α2 human serum glycoprotein, using mixtures of these purified factors. More recently, HSA was shown to decrease the capture of dihydrolipoic acid-coated- quantum dots (QDs) by HeLa cells compared with the no-protein medium (51). The consensus is that indeed native HSA is a dysopsonin for NPs, but this is largely based on studies performed with the purified protein used alone or in combination with few other opsonins. However, no evidence is available on the biologically relevance of serum albumin for NPs in complex media like serum. Nevertheless, presently, HSA is one of the most promising components for effective drug carriers and nanotheranostics (52, 53).

### Complement C3-Derived Opsonins, C1q, MBL, Properdin, IgG, SP-A, and SP-D

It is long known that complement-derived C3 opsonins (C3b/iC3b) are major factors determining the binding of microbial particles to phagocytes in blood, tissues, and clearing organs (liver, spleen, lungs) (54, 55). Experiments dated back to mid-1990s of the past century first clearly pointed that adsorption of C3-derived opsonins on poly(D,L)-lactic acid NPs, likely *via* a Ca^2+^-dependent IgG-triggered pathway (classical pathway), mediates phagocytosis by monocytes (56). Several data show that also PEGylated and many other NPs can activate the complement pathway, leading to C3-derived opsonin deposition mediating their clearance (57–59). This adverse phenomenon may be due to immune recognition of nanomaterial surface portions mimicking microbial or altered cell surfaces, which in turn triggers complement cascade and NP clearance (60). *In vitro* experiments in HS showed the role of complement and of the C3-derived opsonins in phagocyte interaction. Three major lines of evidence are normally used: (1) the sensitivity of pro-opsonic action of serum to chelating agents sequestrating Ca^2+^ (EGTA plus 10 mM MgCl_2_) and so blocking the classical and lectin pathways, or the full block of complement by EDTA which sequestrates both Ca^2+^ and Mg^2+^, so inhibiting also the complement alternative pathway (AP) (61); (2) the use of C3 (and other C proteins) depleted sera, with reintroduction of the purified protein as supercontrol (28, 62, 63); and (3) the neutralizing effect of complement-specific monoclonal antibodies (28, 62, 63). In other cases, selective monosaccharide blockers of differentiated lectin pathways were also used to prevent the association of MBL or ficolins to polymer-coated NPs (62). There is presently a strong record showing that in many instances, it is indeed the antibody-mediated or the innate-triggered complement pathway that leads to deposition of C3b/iC3b opsonins on NPs. These factors seem to actually dominate the effect of other supposed NP alternative opsonins or dysopsonins. Using mouse sera selectively deprived of C factors allowed to demonstrate that dextran-coated superparamagnetic iron oxide nanoworm (SPIO-NW) phagocytosis by mouse peritoneal macrophages *in vitro* is due to collectin MBL-mediated C3b opsonization (63, 64). Tavano et al. (28) showed that C1q, the major collectin-mediating antibody-dependent C activation, can directly bind to PMOXA-coated ORMOSIL NPs leading to C3b/iC3b opsonization and phagocyte endocytosis *via* a Ca^2+^-dependent mechanism. Selective C1q depletion of human serum abrogated C3 opsonization, while purified C1q reintroduction restored C3 opsonization and macrophage capture. Eventually, comparative analysis of the NP proteome after incubation with sera from different individuals revealed a statistically significant positive correlation between macrophage capture and the relative amounts of C3 and other C proteins on NPs. Manipulating the presence of Ca^2+^ and Mg^2+^ selectively blocked C activation, leaving all the rest of the NP proteome almost untouched. Interestingly, clusterin, the major proposed dysopsonin was indeed a major protein in the NP proteome, again showing that the presence of C3b/iC3b in NPs was enough to abrogate any possible dysopsonic action of clusterin. Data support that when C3 opsonin is activated on NPs, it plays a dominant role over clusterin and any other serum protein dysopsonic power.

The direct NP binding by collectin C1q, originally considered only able to mediate antibody or CRP-dependent complement activation, fits with its role also as a direct PRM involved in clearing of microbial or altered self-antigens (64). Consistently, innate recognition by C1q of Gd@C_82_(OH)_22_ NPs, leading to complement activation, was documented in lung cancer-personalized NP proteome (65). The pattern recognition properties of C1q ensured binding also to carbon nanotube (CNT) and triggered complement activation and phagocytosis by macrophages. Recombinant, purified C1q was shown to bind carboxymethyl cellulose multiwall nanotubes (CMC-MWNT) or oxidized multiwall nanotubes (Ox-MWNT) determining a 4- and 1.3-fold capture increase, respectively, by U937 cell line and human macrophages compared with the no-protein medium (66). However, it is strange that the three different globular head monomers were also shown to separately induce an enhanced endocytosis comparable with that of full C1q in CMC-MWNT. C1q also effectively bound to PEG-grafted CNTs, but not on CNTs with adsorbed PEG, without triggering the complement cascade (67). However, in this study, no cellular uptake assays were performed to assess the direct opsonic action of bound C1q.

Properdin also displays direct innate recognition of NPs, a fact leading to potent proinflammatory activation of macrophages. Properdin up-modulates (1.4-fold) the endocytosis of carboxyl-methyl cellulose-coated CNT by a TH-1 macrophagic cell line independently from C activation, a pro-opsonic action which can account that of the whole serum (1.6-fold increase). Such effect is mediated by properdin TSR4 and TSR5 domains, since recombinant forms of these proteins competitively inhibited the effect of native properdin (60% inhibition) (68).

In another study, it was shown that clinical and preclinical NPs are recognized by “natural” pre-existing antibodies and that labeling of NP-bound IgG by C3b/iC3b opsonins is crucial for an effective capture by phagocytes (69). The use of FcR-negative or overexpressing cell lines also indirectly suggested that NP-bound antibodies mediate cell interaction with phagocytes (41, 70).

Importantly, such recognition systems do not totally overlap in humans and closely related preclinical species like the mouse, a fact negatively impacting on nanomedical translation efficacy. Dextran-coated SPIO-NW where opsonized by C3b/iC3b *via* a MBL-triggered lectin pathway, amplified by the alternative (factor B dependent) loop, in mouse serum. Instead, in human serum, the same NPs triggered both lectin and APs, and in some subjects, an IgM-dependent classical pathway, all contributing to C3b/iC3b deposition and opsonization (62). Polymethyloxazoline NPs did not activate C through direct C1q binding in mouse serum as it was in the human serum (28). It is likely that functional divergence characterizes the reaction to nanoformulations in contact with serum from other relevant preclinical models, like the pig, compared with humans. Species specificity of the NP proteome and especially of its immune recognition side emerges as a major critic and still poorly investigated aspect.

Although strong attention is paid to the interaction of NP with serum/plasma proteins, a phenomenon occurring after blood administration, the possible role of biomolecules binding to inhaled particle entering in contact with the bronchoalveolar fluid lining the respiratory mucosae, is also of paramount importance in nanotoxicology and nanomedicine. Also in this case, the host component may influence, NP toxicity, phagocyte clearance and tissue interaction or translocation (71).

Very interestingly, specific innate oligomeric collectins operate in this thin fluid layer: SP-A and SP-D can interact with PAMP or DAMP materials in the lung and also with NPs, favoring their agglutination, phagocytosis, while mediating an anti-inflammatory action (72). Several studies, generally based on protocols testing the effect of purified SPs on the capture of relevant NPs by alveolar macrophages or other phagocytes and APCs, strongly suggest that bronchoalveolar collectins are major innate PRMs influencing the bioactivities of inhaled nanosystems.

McKenzie et al. (73) showed that purified SP-A inhibited the uptake of amine-modified cationic PS NPs by alveolar macrophages, while it favored the uptake of unmodified anionic PS NPs. This is quite a relevant information showing how the same agonist can be judged to be dysopsonic or opsonic depending on the physical-chemical characteristics of the pristine NP surface. Ruge et al. (74) showed that purified SP-A at 10 μg/ml (compatible with BALF SP-A concentration, see (75), mediated the association to alveolar macrophages of magnetite NPs (110–180 nm) coated with different polymers and molecules (starch, carboxymethyldextran, chitosan, poly-maleic-oleic acid, phosphatidylcholine) which was significantly stronger than that observed in the presence of a concentration of BSA (1 mg/ml) indeed much greater than that measured in BALF (76). Mannosilated PEG chains grafted on NPs improved TPH1 macrophage cell capture in the presence of purified SP-A (77). Several metal-oxide NPs when incubated with porcine BALF adsorbed with various efficacy SP-A (78), however no cell-capture experiments were performed. Purified SP-D induced a moderate up-modulation of CMC-CNT phagocytosis, while a symmetrical small downregulation of Ox-CNT phagocytosis, compared with the uptake in the no-protein media (79). This is again a case in which, depending on the nanosurface chemical-physical features, one protein can act either as an opsonin or as a dysopsonin. SP-D (rhSP-D) bound to oxidized and carboxymethyl cellulose (CMC)-coated CNTs *via* its C-type lectin domain and enhanced phagocytosis by U937 and THP-1 cell lines (80).

## Methodolgical Approaches to Identify NP Opsonins and Dysopsonins

### Experimental Criteria

The results discussed in the previous paragraphs reveal the complexity of the biological response to NP proteome formation. Consequently, it is of paramount importance to approach the study of the role of the NP proteome components with a rigorous and well-defined approach, which will necessarily require multiple evidences. Based on the above data, the following criteria are proposed to assess the specific and dominant role of those host proteins which are consistently and reproducibly present in the NP proteome.

#### Statistic Correlation and Informatics Modeling

Significant correlation between the relative abundance of a given factor in the NP proteome formed from different donors’ sera and cell-interaction parameters may be a valuable, although not sufficient *per se*, information to support its functional role in cell interaction. For example, a bioinformatics-inspired multivariate model using the NP proteome fingerprints of a large set of NPs implicated a hyaluronan-binding protein as positive mediators of NP-A549 human lung epithelial carcinoma cell interactions (20). However, NP-cell association of cationic AUT- and MUTA-modified 15-nm gold NPs is only weakly reduced by the presence of high concentrations of free hyaluronic acid as competitive inhibitor (20–25% inhibition), indicating that unidentified hyaluronan-independent mechanisms are prevalent. This corroborates that correlative or statistic relationships alone, although useful to generate working hypothesis, are not enough to assess the role of specific NP proteome components and do not necessarily imply causality.

#### Functional Effect of Purified Components

This evidence may demonstrate the intrinsic opsonic or dysopsonic activity of a given protein, chosen on the basis of its abundance in the NP proteome, its known physiological relevance, or arbitrarily. However, the biological relevance of the tested factor remains to be assessed, since other proteins could play the same role or be functionally dominant. Selected serum proteins (such as albumin, IgG) should be used at concentrations mimicking those present in the body fluids (e.g., HSA, 60 mg/ml; IgG, 7–10 mg/ml). Due to its special composition, where SP-A and SP-D are indeed major protein components, in the bronchoalveolar fluid, the specific role of defined NP-interacting molecules may appear easier to characterize than in serum/plasma. Here, the effect of single purified SPs, shown in several investigations, is therefore a stronger indication of their dominant and biologically relevant role as opsonins or dysopsonins. However, it should also be remembered that the functional contribution of the lipidic component of the surfactant, not always contemplated in these studies, is predicted to be important to modulate SP action on NPs.

#### Depletion

The selective elimination of specific factors, with consequent loss of the effect induced in control host fluids, is a much stronger evidence, compared with the use of the same factors alone. This information can be obtained by immune depletion with specific antibodies or using sera from KO mouse. However, a *super control* based on the reintroduction of the purified protein should be included, whenever possible, to rule out non-specific artifacts due to plasma or genetic manipulations. Here, it should also be noted that accurate functional and proteomic control of the effect of depletion on the rest of the NP proteome should also be performed to evidence possible rearrangements due to the loss of specific proteome components or to the methodology used for depletion. For example, the procedure to delipidate serum from lipoproteins also eliminates clusterin from the NP proteome in ORMOSIL-NPs and abolishes complement activity in serum (Tavano and Papini, unpublished results).

#### The Use of Specific Inhibitors

Specifically recognized inhibitors of innate recognition molecules may be used to ascertain their role in NP binding and endocytosis induction. For example, sugar monosaccharides as *N*-acetyl-glucose and mannose can compete with collectins (62). However, proper control must be performed to exclude wrong mechanistic conclusions and to define the real direct opsonic actor. In particular, comprehensive shot-gun proteomics with quantification of single NP proteome composition should be controlled to exclude secondary recruitment of phagocytosis-active agonists after prevention of a given putative active molecule on NPs. Similarly, the efficacy of the complement cascade and of C3b/c3bi opsonin deposition should be checked after incubation of inhibitors. Neutralizing monoclonal antibodies may also be also valuable tools, as it was shown for anticomplement-specific antibodies.

#### Affecting Cell Receptors for NP-Proteome Components

One indirect way to prove the role of a bound NP protein could be the downregulation of specific receptors, for example, by RNA interference or gene mutation or their overexpression due to transfection procedures in appropriate model cells. Also, receptor neutralization by specific antibodies or competition with protein domains is applicable. Again, this may be a useful additional evidence to support the specific action of a single proteome component.

### Minimizing Ambiguities in Attributing Opsonin or Dysopsonin Properties to NP-Bound Proteins

**FIGURE 4:**
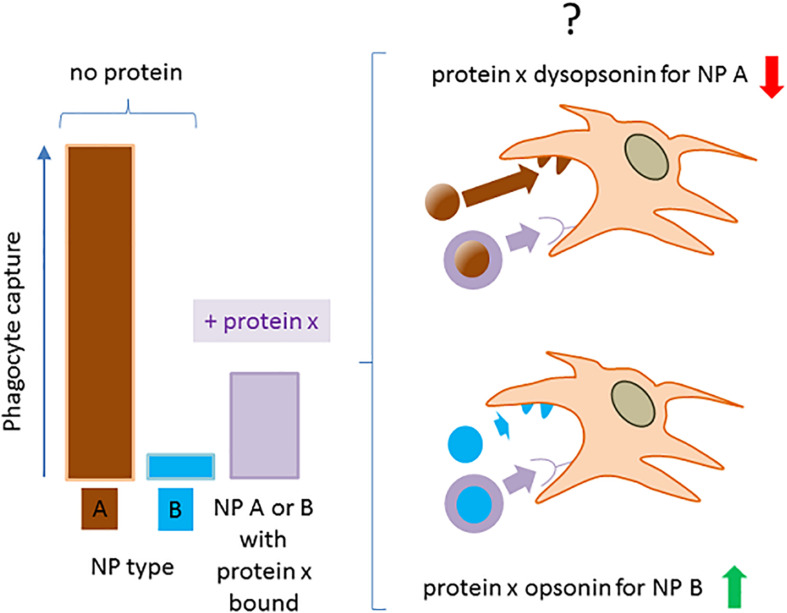
The operative definition of a NP-bound protein as opsonin or dysopsonin may be biased. The scheme summarizes how NPs with a very different ability to be captured by phagocytes in the absence of proteins, can be differently modified by the same protein, leading to a controversial classification of its opsonic or dysopsonic action.

Available data suggest the convenience of refocusing in more detail on the very notions of dysopsonin or opsonin in the nanofield, generally assumed as self-evident. If we look at literature, we may in fact sometimes assist conflicting conclusions on the intrinsic pro- or antiphagocytic activity of defined proteins. Excluding trivial experimental non-reproducibility, part of such discrepancies may stem from the way we measure opsonin/dysopsonin activity and on our limited perspective on the models used. Indeed, the classification of a given host protein binding to NPs as an opsonic or a dysopsonic agent may be ambiguous if only based on the relative cell capture of NPs in the presence of this single protein, compared with the NP capture in the no-protein media. In fact (as exemplified in Figure 4), the intrinsic ability of the new nanohost interface to regulate phagocyte internalization could be higher, lower, or equal to the one expressed by the nude original particles, depending on their pristine chemical composition. For example, nothing prohibits that a protein may decrease the intrinsic binding to cells and phagocytes of a highly interactive nanomaterial, and that, on the contrary, the same protein could increase the binding of another, intrinsically more inert material. Moreover, different materials could induce diverse conformational modifications or denaturation processes of the same protein, which may result in changes of its cell-binding efficacy.

**FIGURE 5:**
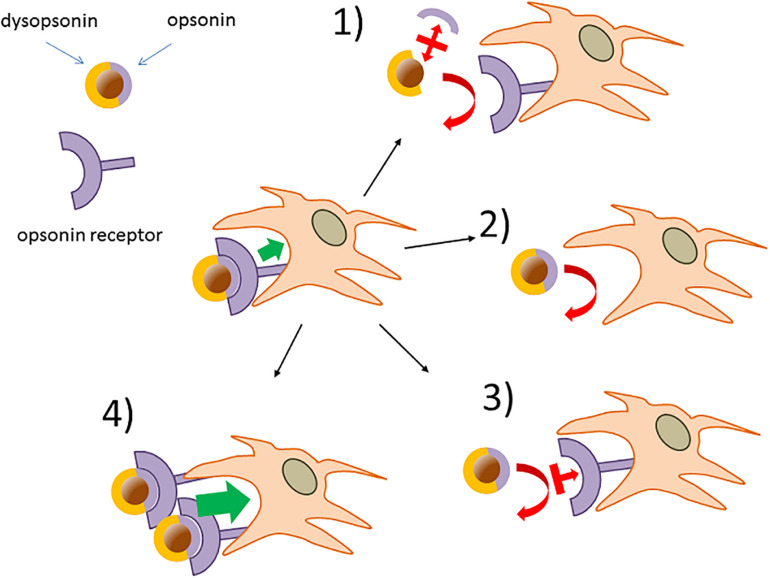
Verification of opsonins and dysopsonins based on the existence of specific receptor-mediated cell-internalization mechanisms. Phagocytes may express NP opsonin receptors (*e.g.*, FcR) responsible for NP capture. (1) The functional role of hypothetical NP-bound protein as an opsonin can be tested using factors with selective displacing efficacy (*i.e.*, chelating agents, antibodies, inhibitors), leaving the dysopsonin associated to the NPs. (2) Deletion or downregulation of the hypothetical opsonin receptors can be used to provide additional evidence on NP opsonins. (3) Validated neutralizing antibodies or competing inhibitors affecting the opsonin receptor action or (4) upregulation of opsonin receptors are additional corroborating possible approaches.

A situation exemplifying such case is present in early studies by Thiele et al. (50), where the cell capture of microparticles (1 μM) with different charges and surface properties was measured in the no-protein media or in the presence of three selected serum proteins. All proteins tested (α2 human serum glycoprotein/α2 GP, IgG, and HSA) decreased the strong capture of cationic particle compared with the no-protein medium, so apparently acting as dysopsonins. However, when more hydrophobic and less capture-prone particles where used, α2 GP and IgG improved particle capture compared with the no-protein medium, while HSA still acted as a strong dysopsonin, also able to compete with α2GP and IgG opsonic action. This clearly indicates that one protein could look like opsonic or dysopsonic depending on the characteristic efficacy of the pristine material to interact with cells and suggests that the comparison with cell-capturing efficacy in the no-protein media to classify a protein as dysopsonin or opsonin could be sometimes misleading. Hence, it may be much more biologically relevant and pharmacokinetically predicting to define the possible action of a given protein based on the measurement of specific ligand/receptor interactions, favoring endocytosis/phagocytosis. In such case, what is relevant is the comparison of the NP–cell interaction and the consequent cell endocytosis in controls and in samples where, due to experimental manipulation or natural situations, the well-delineated receptor-mediated effect is inhibited or absent. Such comparison is less prone to bias if it is performed in more physiological conditions allowing also the binding of other proteins (such as full serum, BALF) (e.g., annihilation of C3b-C3b receptor complex formation by divalent ion deprivation to block complement in serum). Therefore, it may be safer and less prone to bias to define an opsonin as a molecule able to mediate the binding of a given NP to cells through a defined, and possibly identified, receptor (Figure 5). Consequently, the biological relevance of such phenomenon, within the general context, may be tested *in vitro*, by assessing its relative effect compared with the efficacy of cell capture after its experimental selective inhibition.

## Opsonin-Dysopsonin Balance on Nanoparticles and Its Tilting by Complement

**FIGURE 6:**
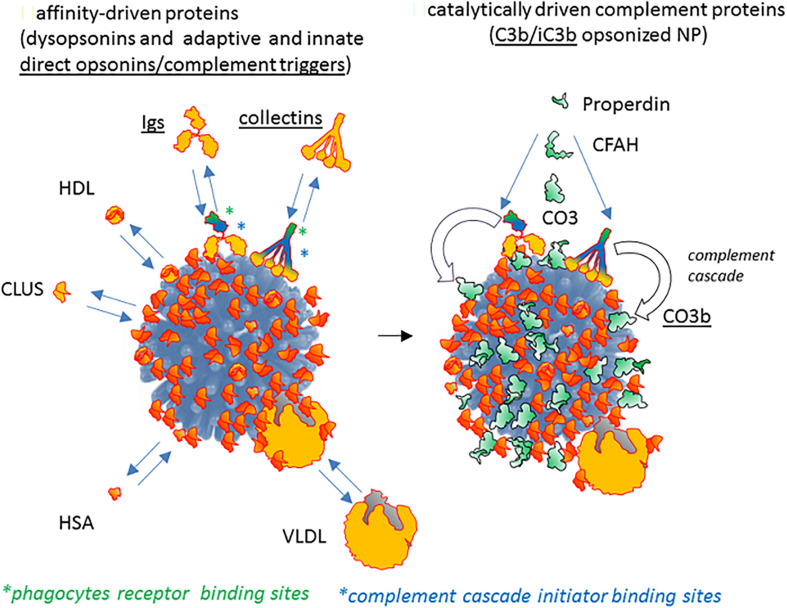
The contribution of passive host proteins binding and of active complement cascade in modeling opsonin/dysopsonin balance and the final phagocyte capture of NPs. Present literature suggests that some host proteins potentially able to bind NPs **(left)** are dysopsonin since they do not mediate the binding of the NP to receptors and clearing by macrophages or monocytes (*e.g.*, HSA, clusterin, HRG). On the contrary, other proteins (in general, belonging to the adaptive or innate immune system, like immunoglobulins, collectins, properdin, surfactant proteins) do bind to phagocyte-expressed receptors, being potential opsonins. Both molecule classes (dysopsonins and direct opsonins) associate to NPs thanks to a chemical equilibrium regulated by their affinity/avidity for determined chemical features of the NP coats. The **right** panel shows how the immune agonists recognizing the NP surface as antigenic or as microbial/altered self danger signals not only can be directly opsonizing but can also trigger the enzymatically driven complement cascade by activating protease transductors, like C1 or MASPs, eventually leading to a C4-dependent generation of C3b/iC3b major opsonin, covalently fixed on the NP surface.

With the above caveat, recent literature broadly indicates the following scheme of NP-host serum/BALF effect on phagocytosis. A pristine material, either nude or derivatized with supposed stealthing polymers, is in general phagocytosed less, although not necessarily depending on the material type, when major dysopsonins associate at the host interface (e.g., clusterin or HSA). However, the parallel binding of specific innate or adaptive immune proteins can generally overcome the protective effect of clusterin or other dysopsonic serum proteins by acting as direct opsonin or by activating the complement cascade leading to the deposition of the major opsonin C3b/C3bi. In such scenario (depicted in Figure 6 and discussed in detail in the relative legend), the whole spectrum of variable phagocytosis efficacies mediated by different pristine coatings with differentiated intrinsic direct cell-binding mechanism “collapse” to a narrower and more reduced capture efficacy range. This may happen, for example, if host factors like clusterin or HSA, with similar shielding and stealth efficacy, bind similarly to a wide range of NPs. However, as we have pointed out in this review, in physiological conditions, part of the residual surface not engaged with dysopsonins may bind antibodies or innate PRMs, directly acting as opsonins or amplifying complement-mediated opsonin deposition. Such superimposing phenomenon may be as well modulated by specific and differential nanosurface determinants or by an exclusive or synergic modulation by bystander-bound host proteins. It is relevant to notice that both C4 and C3 opsonin deposition on NPs are modulated by the chemical reactivity of their internal thioester bonds with –OH and –NH_2_ groups or other nucleophiles expressed by host-exposed NP components (e.g., polymer coats) or by bystander NP-associated proteins. Hence, the final opsonization efficacy may be further increased, depending not only on the extent of initial complement triggers of NP binding (an exquisitely thermodynamic equilibrium step) but also on the overall chemical reactivity of the NP coats and NP-bound protein set, which will concur to determine the extent of C3b fixation available to C3 receptors on NP-clearing phagocytes. The chemical reactivity of NP coats and bound proteins is expected to be especially relevant in those cases in which C3b fixation results from genuine AP activation, since this occurs in the absence of initial triggers like antibodies or lectins. Moreover, C3b opsonization may be further modulated by the NP efficacy in favoring or interfering with the association of complement-regulating components, such as C4b-binding protein A (C4BP), the complement factor H (CFH), or factor H-related (FHr) proteins. Overall, present literature suggests that the effective complement cascade activation on NPs can strongly unbalance the initial opsonin/dysopsonin proportions, becoming in several cases a major functional actor in regulating the stealth features of NPs. More in general, we can predict that, due to the above variables, opsonins may be differently added to NPs, resulting in a wide spectrum of phagocytosis up-modulation: from almost zero (dysopsonic action prevailing) to moderate effect (opsonins and dysopsonins balanced and reciprocally neutralizing) or, at the opposite extreme, to strong capture when opsonin density overcomes and neutralizes dysopsonin effects.

## Concluding Remarks

**FIGURE 7:**
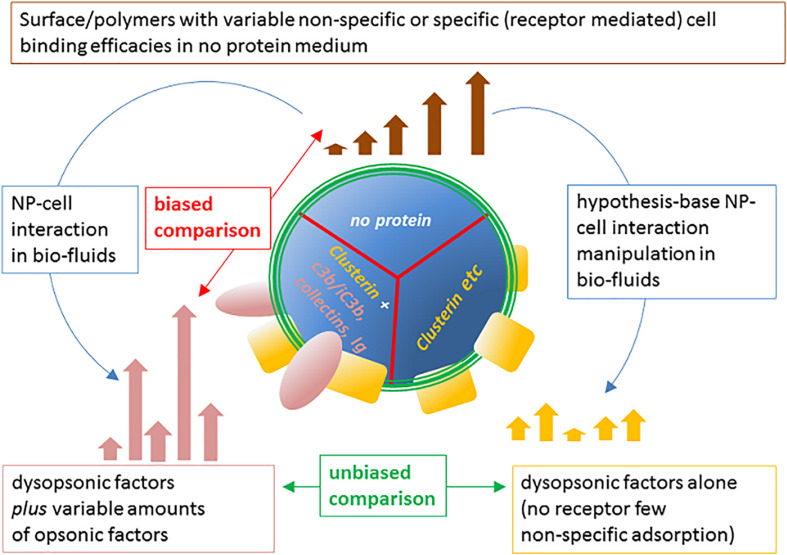
Modulation of NP cell-capture efficacy spectrum of the pristine material surface by host NP proteome and proper approach to identify opsonins. The intrinsic ability of NP chemical coatings to mediate the internalization by phagocytes can vary significantly, depending on charge, hydrophobicity, or other specific characteristics of the coating agents and polymers. The resulting spectrum of clearance efficacy **(top)** of possible surfaces in the no-protein medium is modified by the possible binding of host proteins to NPs in biofluids **(left)**. This may result in up-modulation, down-modulation, or non-modulation of one defined NP cell capture compared with the one in the no-protein medium. However, the selective hampering of opsonins in complex medium, differently modulates the capture spectrum **(right)**, allowing to evaluate the capture efficacy due to remaining dysopsonins. This comparison allows to unequivocally define the biological relevance *in vitro* of the identified specific opsonic factors and obtain relevant insights on the molecular mechanisms involved.

To conclude, as summarized in Figure 7, it is evident from literature that both nude and host protein-modified NPs are characterized by differential phagocytosis ranges, where the ranking can be rearranged. Such phagocytic rates, in general, collapse into a narrower, and tendentially reduced, capture spectrum efficacy in the presence of biofluid, naturally or artificially deprived of pro-opsonic agents. However, since in natural conditions the no-protein medium is not present, the most critic comparison to spot factors improving RES clearance and possibly affecting a nanoformulation half-life in blood, is between control conditions (normal host fluid normal cell acceptor) with manipulated host fluid or cell acceptors, highlighting the determinant role of specific host NP-bound proteins and specific receptors on acceptor cells.

Once such biochemical phenomenon is delineated, and its assay developed and standardized, a feedback iterative loop may be applied to test coating designs lacking the opsonin deposition, for better stealth nanoformulations.

## Author Contributions

All authors equally contributed to text writing and figure assembly.

## Conflict of Interest

The authors declare that the research was conducted in the absence of any commercial or financial relationships that could be construed as a potential conflict of interest.

## Abbreviations

MS: mass spectrometry
HRG: histidine-rich glycoprotein
HSA: human serum albumin
PMNGs: polymorphonuclear granulocytes
PLA: poly(D,L-lactic acid)
PLGA: poly(lactic-co-glycolic acid)
PCL: poly(varepsilon-caprolactone)
PAMP: pathogen-associated molecular patterns
DAMP: damage-associated molecular patterns
VLDL: very low-density lipoprotein
SP-D: surfactant protein-D
DMBT1: deleted in malignant brain tumor 1
Kin-1: kininogen 1
C1q: complement factor 1 q
IgM: immunoglobulin M
HDL: high-density lipoprotein
BALF: bronchoalveolar lavage fluid
PAP: pulmonary alveolar proteinosis
SRCR: scavenger receptor cysteine rich
HMWK: high molecular weight kininogen
LDL: low-density lipoproteins
IgG: immunoglobulin G
FCS: fetal calf serum
PEG: polyethylene glycol
ORMOSIL: organically modified silica
PS: polystyrene
HES: hydroxyethyl starch
C3: complement factor 3
PMOXA: polymethyloxazoline
QD: quantum dot
MBL: mannose-binding lectin
HS: human serum
EGTA: ethylene glycol tetraacetic acid
EDTA: ethylene diamine tetraacetic acid
AP: alternative pathway of complement activation
CNT: carbon nanotubes
CMC-MWNT: carboxymethyl cellulose multiwall nanotubes
Ox-MWNT: oxidized multiwall nanotubes: FcR, receptor of the Fc fragment
SPIO-NW: superparamagnetic iron oxide nanoworms
AUT: 11-amino-1-undecanethiol
MUTA: 11-mercaptoundecyl tetraamine
α 2 GP: alpha 2 human serum glycoprotein
PRM: pattern recognition molecules
C4: complement factor 4
C4BP: C4b-binding protein
CFH: complement factor H
FHr: factor H related.
